# Liquid and Solid Embolic Agents in Gonadal Veins

**DOI:** 10.3390/jcm10081596

**Published:** 2021-04-09

**Authors:** Francesco Tiralongo, Giulio Distefano, Monica Palermo, Antonio Granata, Francesco Giurazza, Francesco Vacirca, Stefano Palmucci, Massimo Venturini, Antonio Basile

**Affiliations:** 1 Radiology Unit I, Department of Medical Surgical Sciences and Advanced Technologies “GF Ingrassia”–University Hospital “Policlinico-San Marco”, University of Catania, Via Santa Sofia n° 78, 95123 Catania, Italy; giuliodistefano@gmail.com (G.D.); monica.palermo91@gmail.com (M.P.); f.va77@libero.it (F.V.); spalmucci@unict.it (S.P.); basile.antonello73@gmail.com (A.B.); 2 Nephrology and Dialysis Unit, “Cannizzaro” Hospital, 95123 Catania, Italy; antonio.granata4@tin.it; 3 Interventional Radiology Department, Cardarelli Hospital of Naples, 80131 Naples, Italy; francesco.giurazza@aocardarelli.it; 4 Department of Diagnostic and Interventional Radiology, Circolo Hospital, Insubria University, 21100 Varese, Italy; massimo.venturini@uninsubria.it

**Keywords:** varicocele, pelvic congestion syndrome, embolic agent, vein embolization, interventional radiology

## Abstract

Male varicocele and pelvic congestion syndrome (PCS) are common pathologies with high predominance in young patients, having a high impact on the quality of life and infertility. Lately, the use of different endovascular embolization techniques, with various embolizing agents, shows good technical results and clinical outcomes. With the aim of presenting the “state of the art” of endovascular techniques for the treatment of male varicocele and PCS, and to discuss the performance of the different embolic agents proposed, we conducted an extensive analysis of the relevant literature and we reported and discussed the results of original studies and previous meta-analyses, providing an updated guide on this topic to clinicians and interventional radiologists. We have also underlined the technical aspects for the benefit of those who approach this type of interventional treatment. Our review suggests promising results in both the endovascular embolic treatment of male varicocele and PCS; for varicocele, a success rate of between 70% and 100% and a recurrence rate of up to 16% is reported, while for PCS it has been found that technical success is achieved in almost all cases of endovascular treatment, with a highly variable recurrence rate based on reports. Complications are overall rather rare and are represented by periprocedural pain, migration of embolic media and vascular perforations: severe adverse events have been reported very rarely.

## 1. Introduction

Scrotal varicocele in men and pelvic congestion syndrome (PCS) in women represent two relatively frequent pathological conditions in the young-adult population, with important implications on quality of life and a significant impact on fertility. In these two conditions, gonadal venous vessels are abnormally dilated (sometimes secondary to other causes) and flow is slow and retrograde in the gonadal vein.

Traditionally, the resolutive treatment of these pathologies was based on surgery, but in recent decades interventional radiology has taken hold on this topic: there are several reports with large case series and various meta-analyses that demonstrate that, overall, transcatheter endovascular treatments are (at least) not inferior to the surgical approach, both in terms of technical and clinical success, even after prolonged follow-up, and that complications are relatively rare [1].

The aim of this study was to illustrate the main embolic agents used in the percutaneous treatment of male varicocele and pelvic congestion syndrome, evaluating their mechanism of action, the technical differences of use, the complications and the technical success rate and the relapse rates for each embolic agent.

We conducted an extensive analysis of the relevant literature through the PubMed and Google Scholar databases, re-evaluating previous meta-analyses, guidelines, original studies and case reports with the aim of illustrating the role of various embolic agents in male and female pathology and of providing an updated guide on this topic to clinicians and interventional radiologists. We reported the main clinical and instrumental features of scrotal varicocele and PCS and proposed a review of embolic agents, commenting on their indications, technical aspects, expected outcomes and possible complications in adult patients.

### 1.1. Male Varicocele

Male varicocele (MV) is an abnormal distension (enlargement) of the pampiniform plexus caused by reversed blood flow and/or impaired drainage of the testicular or internal spermatic vein (ISV) [2].

The incidence of varicocele in young healthy male individuals is 8–23%; it involves the 40% of infertile males [3,4]. The etiology of male varicocele is multifactorial and there are three main theories to explain the onset of varicocele: (1) the left internal spermatic vein inserts into the left renal vein at an angle of 90° and this angle leads to a higher hydrostatic pressure of ISV; (2) the congenital and/or acquired lack of functioning valves in ISVs which leads to reflux of blood; (3) the compression of the left renal vein (LRV) between the aorta and the superior mesenteric artery (SMA), also known as nutcracker phenomenon [5,6]. The left side is involved in 75–95% of cases, while the right side in only 5–10% of cases; varicocele is bilateral in 1–15% of cases [6].

Male varicocele is associated with alteration of sperm count, motility and morphology, leading to mild and moderate oligospermia, teratospermia or astheno-teratospermia [7].

With regards to symptoms, MV is often asymptomatic; local pain (in testicle, scrotum, or groin), varying from sharp to dull discomfort, scrotal heaviness and testicular volume loss are also present in about 2% to 10% [8].

Treatment is indicated in: symptomatic (painful) and palpable (grade 1–3) varicocele, in particular, persistent scrotal pain is an indication for repair, regardless of fertility status [2]; in a subclinical scenario, varicocele repair is not indicated as it does not improve spermatic parameters and does not increase the chance of spontaneous pregnancies [9,10]; in couple infertility, varicocele treatment has proven to be effective in men with oligospermia and unexplained infertility [11] and provides a good opportunity for natural conception [2].

Treatment consists of interruption of reflux through the ISV and its branches superior to the pampiniform plexus. This can be achieved by surgical or percutaneous endovascular techniques [12,13,14,15]. 

Varicocele endovascular embolization was first proposed in the 1970s and is equivalent to the surgical ISV clipping: with the Seldinger technique, through a neck, groin or arm approach, a diagnostic catheter reaches the renal vein for diagnostic phlebography. After venography demonstrates ISV dilatation and the presence of persistent collateral veins, ISV is gained and the embolic agent is directly delivered [16,17].

The procedure is performed on an outpatient basis under local anesthesia on a tilted X-ray table [15,18]. The presence of collateral veins is the major anatomical factor contributing to treatment failure [19]. Radiologic treatment offers the advantages of causing less patient discomfort and rapid recovery in comparison to the more invasive approach of varicocelectomy [20]. Endovascular treatment offers a lower cost and a lower recurrence rate than surgery and prevents the incision and splitting of the abdominal muscles [14,21,22,23,24].

In addition, surgery does not provide the possibility to visualize the exact varicocele anatomy and collaterals [25]. Concerning open varicocelectomy techniques, high recurrence and complication rates have been reported, with complications ranging from hydrocele formation, testicular artery injury, epididymitis, and vas deferens occlusion, which are otherwise rarely seen in the endovascular approach [26].

### 1.2. Pelvic Congestion Syndrome

Pelvic congestion syndrome (PCS) is a pathological condition characterized by chronic pelvic pain (CPP, defined as pain lasting at least six months associated with symptoms indicating gynecologic, lower urinary tract, bowel, and pelvic floor dysfunction) and retrograde flow in ovarian veins, which appear dilated, and para-uterine varices [27,28]. CPP is associated with pelvic varicocele in about 30% of cases, particularly in pre-menopausal multiparous women with no other known causes of pelvic pain [28].

This condition can be primitive, because of the lack of valves (15% of cases) or of the presence of incompetent valves (up to 40% of cases), monolaterally or bilaterally [29], or it may be secondary, due to extrinsic compression of upstream venous vessels, as occurs in anterior or posterior Nutcracker syndrome (for the LRV) entrapment [30] or in May–Thurner syndrome (for the left common iliac vein compression) [31].

The diagnosis is made on the basis of the clinical features and imaging tests.

Clinically, PCS is associated with a feeling of heaviness, that can be exacerbated during menstruation and pregnancy, by coitus (dyspareunia) or by physical activity; gastrointestinal disorders, bladder irritability and menstrual disorders may also be present [32]. Angiographic findings include gonadal vein reflux, dilatation of gonadal, uterine and utero-ovarian vein (diameter greater than 5 mm), contralateral reflux, opacification of vulvar varices, and a reno-caval gradient of >4 mmHg [33]. In order to stage the pathology through the score system proposed by Beard [34], the maximum diameter of the ovarian vein, timing of disappearance of contrast medium, and the degree of congestion should be carefully evaluated.

Indications for treatment include pelvic varicosities associated with clinical feature of PCS, symptomatic labial/perineal varicosities, lower-limb varicosities with atypical distribution or which recur immediately after treatment, and unexplained CPP in patients with varicosities.

The treatment of PCS includes medical, surgical and interventional radiology techniques; the latter have proved to be not inferior to traditional surgery, with the benefit of being less invasive, less expensive and better tolerated by the patient [35]. Secondary forms due to narrowing of LRV can be treated with stent placement at the stenotic tract [30].

## 2. Embolic Agents

Embolic agents can be classified on the basis of their physical state (solid vs. liquid), mechanism of action (mechanical vs. chemical), origin (autologous vs. synthetic) and on the duration of occlusion (temporary vs. permanent) [36]. In this narrative review, we focus on the different physical state of embolic agents used in gonadal veins (Table 1).

### 2.1. Solid Agents

Solid embolic agents include devices that provide mechanical embolization; they are distinguished in coils, vascular plugs and balloons.

In 1975, Gianturco et al. [37] introduced the use of coils for embolotherapy; over the years, different configurations have been proposed, with a wide variety of shapes (straight, helical, spiral and 3D shapes) and sizes (the length can range from 1 to 300 mm and the diameter from 1 to 27 mm). In order to increase the thrombogenity, they can be coated with different materials like Dacron, nylon or silk fibers. Coils can be delivered by pushing or by using a specific detachment system. While pushable coils are less expensive and allow a quicker procedure, using detachable coils make their placement more accurate and predictable. It should be recalled that the effectiveness of the coil embolization depends on the patient’s coagulation state [38].

The Vascular Plugs (AVPs) are permanent mechanical embolic devices. They are disks of self-expanding nitinol mesh and they are suitable especially in high-flow vessels, providing a lower risk of migration than coils. Different types of AVPs present different morphology and sizes, each one fitting with specific application. Releasing an AVP is relatively easy for the operator, thanks to the delivery wire that is associated to a stainless-steel micro screw, which allows the radiologist to retrieve and reposition the plug before the final release, that is obtained by rotating the wire with a torque device. AVPs are relatively expensive but can significantly reduce procedure time. Like coils, AVPs depend on a patient’s coagulation status to obtain a successful embolization [38,39].

Detachable balloons are mechanical embolic devices [40,41]. The balloon should be chosen slightly larger than the diameter of the vessel to be occluded and they are attached to their delivery catheters and, if it is large enough, two balloons can simultaneously be advanced through it into the vessel in order to reduce the risk of premature detachment. The proximal balloon is used to arrest the flow and removed after the distal balloon is deployed—its use is nowadays not common.

### 2.2. Liquid Agents

Liquid embolic agents include tissue adhesives, also known as glues (N-butyl-cyano-acrylate, NBCA, or N-butyl-cyano-acrylate + methacryloxysulfolane, NBCA-MS) and sclerosing agents prepared as foam or liquid (e.g., polidocanol, sodium tetradecyl sulfate, sodium morrhuate, or ethanolamine oleate), the latter often used in combination with coils and/or balloons (sclero-embolization).

Cyanoacrylate is a glue with a high adhesive strength, approved by the Food and Drug Administration (FDA) in 1998 for trauma and surgery injuries [42,43]. After contact with blood, it starts a polymerization that triggers an exothermic reaction that contributes to the damage of the vascular endothelium. Cyanoacrylate has a dual mechanism of action: as an embolic agent and as a sclerosing agent [22]. There are several chemical forms, depending on the chemical chain (methyl, ethyl, n-butyl, isoamyl, isohexyl, and octyl cyanoacrylates) [44].

Cyanoacrilate is radiolucent, so it should be mixed with Lipiodol and released under fluoroscopic control. The mixture with Lipiodol also modulates the polymerization rate [45]. As reported in literature, a mixture at a ratio of 1:1 allows to achieve a rapid polymerization that avoids migration [19,45]. Classic NBCA (Histoacryl^®^, Braun, Melsungen, Germany) has a polymerization temperature of 90 °C and a polymerization time rate < 30 s. In 2001, a new tissue adhesive system came on the market as NBCA-MS or N2BCA (Glubran^®^2, General Enterprise Marketing, Viareggio, Lucca, Italy) [46]. It is composed by two adhesives, consisting of the same monomer as NBCA (n-butyl-2-cyanoacrylate) with the addition of a co-monomer (methacryloxysulfolane), and a different way of polymerization [14,47]. With Glubran2, the polymerization rate is slower, and the handling and releasing are easier. The exothermic reaction is weaker (45 °C), resulting in lesser inflammation and histotoxicity than when Histoacryl^®^ is used, and therefore it is less painful at the time of injection. The final polymer is more flexible than conventional cyanoacrylate. Glubran2 has a bacteriostatic effect and does not polymerize in contact with air; it is the only glue with a CE-mark for endovascular use [14,15,19,45].

Vanlangenhove et al. tested Histoacryl^®^ and Glubran^®^2 in a double-blind, prospective, randomized study: they found that both glues can be handled in the same way and that the embolic result is similar [15]. 

Sclerotherapy is a powerful and commonly used embolic technique; it refers to the introduction of a foreign substance into the lumen of a vessel, aiming to create venous wall damage leading to occlusion of the vessel [48].

Sclerosing agents work by causing irreversible damage to the venous wall by attacking lipids and cellular walls—the inflammatory response is a result of cell damage with fibroblast proliferation that leads to sclerosis. In addition to fibrosis, agents may produce other effects such as thrombosis, extraction of proteins from lipids, denaturation of proteins, cell dehydration by osmosis, and physical obstruction by polymerization [49]. Various sclerosing agents have been described, including osmotic agents, detergents, chemical agents, and erosive agents. In particular, detergents are used in gonadal veins embolization. Detergents act by disrupting cell membranes through the mechanism of protein theft denaturation. Endothelial damage occurs within minutes of the administration of these agents and can spread farther from the injection site. The advantages of detergents are that their concentrations can be adjusted to match the size and type of vessel being treated and they can be made into foam. Common detergent sclerosants include polidocanol (Aetoxysclerol^®^ 1-2), sodium tetradecyl sulfate (STS), sodium morrhuate, and ethanolamine oleate. Frequently, sclerotherapy is performed by mixing air or oxygen into the sclerosant, known as foam sclerotherapy [50].

A particular liquid agent is Onyx^®^ (ev3, Irvine, CA, USA), a liquid non-adhesive embolic agent, also known as ethylene-vinyl alcohol (EVOH). Onyx is a biocompatible copolymer dissolved in dimethyl sulfoxide (DMSO) that, when in contact with blood, solidifies to a rigid cast, as a kind of plastic, and can be then pushed and extended in different directions [51,52].

## 3. Clinical Results in Male Varicocele

Male varicocele embolization can be achieved using solid and liquid embolic agents. Solid embolic agents for treatment of male varicocele embolization include coils, detachable balloons, vascular plugs.

**Figure 1:**
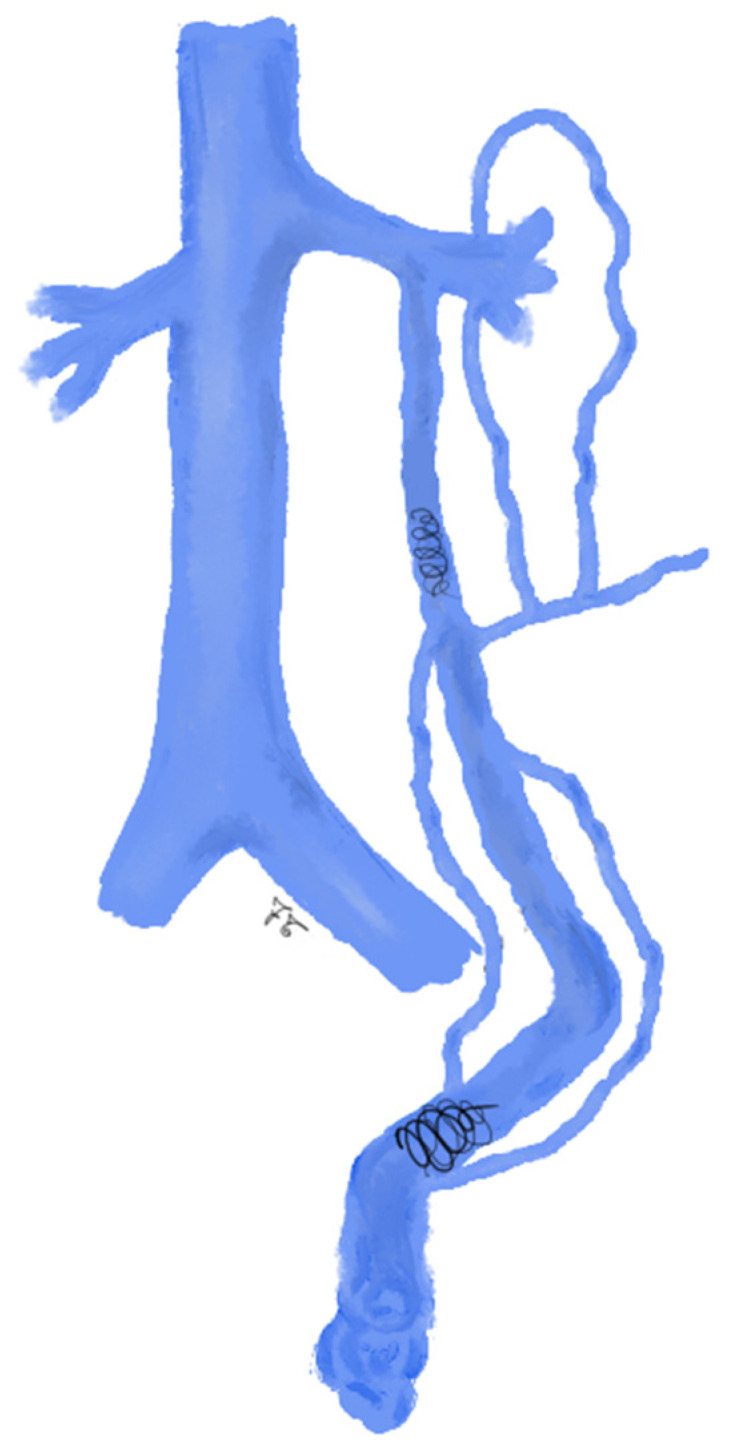
Drawing shows spermatic vein embolization with coils: coils are first deployed as distal as possible and up to the inguinal canal. Then, a sandwich occlusion of the spermatic vein is performed with additional coils in the proximal part of the spermatic vein.

More than 30 years ago, coils were introduced by Thelen et al. [53] and they showed to be effective in varicocele treatment. Coils-embolization is the most commonly used technique, due to coils simplicity in handling and availability [54]. Initially, the original 0.038 inch Gianturco coils were used; subsequently, fibered stainless-steel coils and platinum coils were developed, ranging from 0.035 to 0.038 inches and oversized up to 20%. Coils can be pushable or detachable, the latter improve correct placement [53]. During embolization procedures, coils are first deployed through the microcatheter as distal as possible and up to the inguinal canal. Then, a sandwich occlusion of the spermatic vein is performed with additional coils in the proximal part of the spermatic vein (Figure 1) [45]. The coils deployment is carried out during Valsalva maneuver in order to have the maximum diameter of ISV. The main disadvantage of coils is that they are not as effective as the surgical clipping if collateral vessels are present [25,53,55]: this can lead to the recanalization of the varicocele [45].

Coil embolization has been shown to lead to several complications; in particular, epididymo-orchitis, pampiniform plexus phlebitis or hydrocele were the most common complications, observed in 3.4% of the patients [56]. An episode of femoral phlebitis was reported and treated on an outpatient basis [57]; moreover, one episode of coil migration in right atrium was reported: coil was immediately captured and retrieved [56]. Coil migration may cause pulmonary embolism, but this kind of complication is almost nil thanks to the development of detachable coils [45]. Bilrerio et al. reported a rate of complication of 0.97% with coils, significantly lower than the 9.7%, reported by Bechara et al. [20,58]. Makris et al., in a systematic review including 898 patients treated with coils and 1628 patients treated with a combination of coils and sclerosing, reported a mean technical success rate of 92% for both coils group and coils + sclerosing group, with an average relapse rate of 9.1% for coils group and 8.44% for combined treatment (without significant difference between the two groups) [56].

A further factor to be evaluated in coil embolization is the failure rate due to technical fault, ranging from 3 to 28% [15]: this issue is related to the difficulty in catheterizing the spermatic vein in case of aberrant vessels, valves, vasospasm and intimal dissection [53,59,60].

Amplatzer vascular plugs may be used as an alternative to coils [61]; both plugs and coils were used to ensure complete occlusion of the spermatic vein through the sandwich technique. In cases of large veins, both plugs and coils were used in combination to ensure complete occlusion of the spermatic vein [45].

Detachable balloons can be navigated through an introducer catheter and detached in the ISV. Depending on the size of the varicocele and on the presence of collateral vessels, one or more 1 or 2 mm detachable balloons can be used. Balloons are frequently used in combination with other agents, like coils or sclerosing agent-like sandwiched 70% dextrose [18].

Liquid agents, despite solid agents, have the advantage of being able to penetrate into the collateral venous network around the ISV.

Tissue-adhesives (Cyanoacrylates) were first introduced in varicocele treatment by Kunnen et al. in 1980. The first used glue was a mixture of contrast agent and isobutyl-2-cyanoacrylate, IBCA (Bucrylate, Ethicon) [21]. Due to its possible carcinogenicity, IBCA was later replaced by NBCA (n-butyl-2-cyanoacrylate or Embucrilate; Histoacryl Transparent, Braun, Tuttlingen, Germany), which, however, never received a European conformity (CE) label for intravascular use, followed by Glubran 2—the only glue with a CE-mark for endovascular use [62,63,64]. Glue should be injected immediately after an injection of an anionic solution (5–10% dextrose solution) in order to fill the catheter dead space and avoid glue polymerization into the catheter lumen [58].

**Figure 2:**
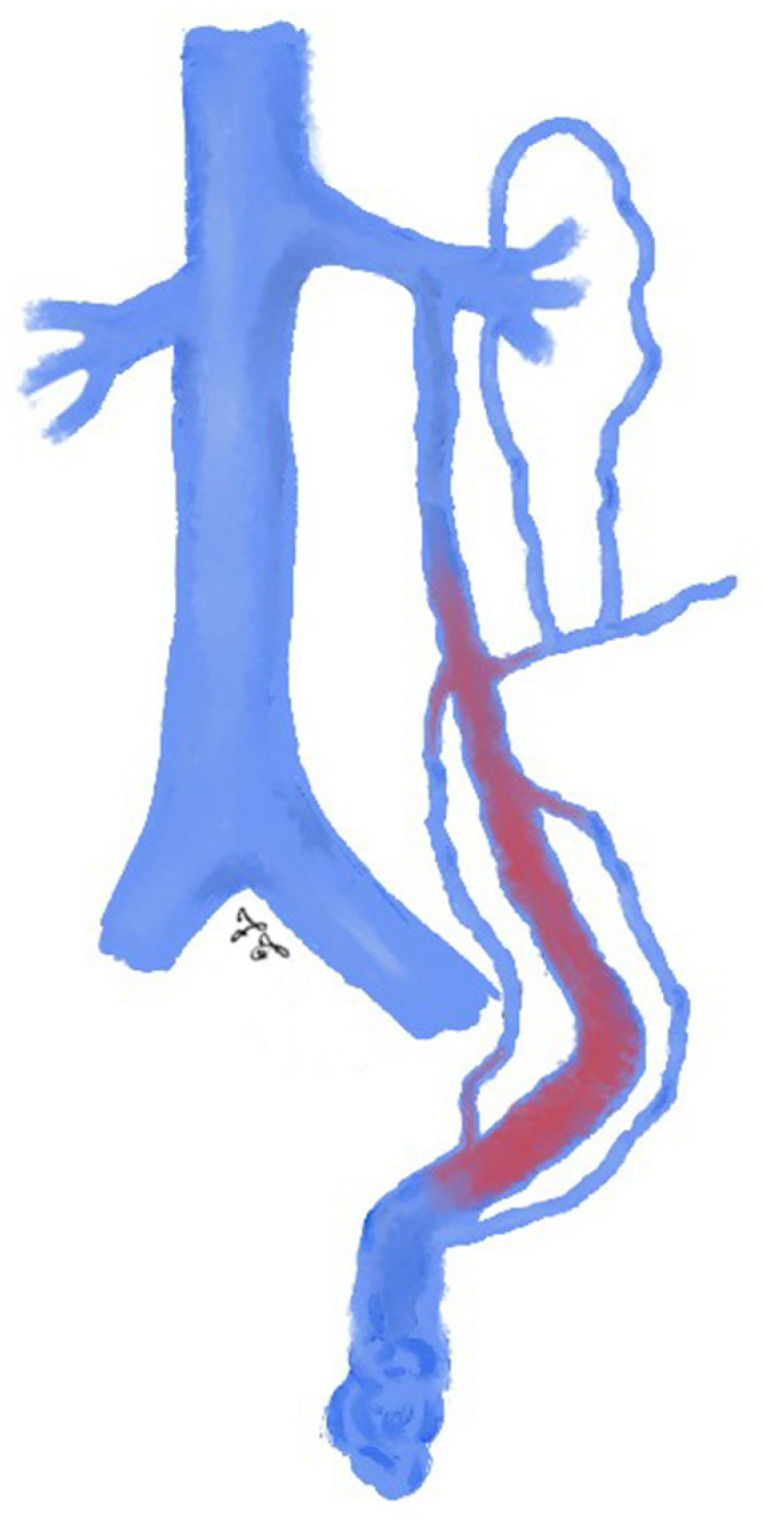
Drawing shows spermatic vein embolization with glue: glue is sequentially injected and pushed into the distal intrapelvic segment of the gonadal vein as well as into the collaterals; injection should be stopped before the pampiniform plexus is reached.

The glue is sequentially injected through the microcatheter and pushed into the distal intrapelvic segment of the gonadal vein as well as into the collaterals; the catheter should be withdrawn while injecting the glue under fluoroscopy guidance. Injection should be stopped before the pampiniform plexus is reached (Figure 2) [45]. As soon as the glue comes in contact with blood, the polymerization process starts, and a permanent occlusion of the vessel occurs [15]. As already mentioned, liquid agents, unlike non-liquid ones, have the advantage of affect also the collateral pathways, thus increasing the effectiveness of the procedure [26,65].

Cyanoacrylate glue for varicocele embolization was shown to be a safe and effective alternative to coils [58]; in particular, the mean procedure time was shorter with glue, resulting in a lower radiation (shorter duration of scopy, less kinetic energy released per unit mass, lower DAP) [45]. Embolization with glue is also cost-effective: one cubic centimeter has a comparable cost of single conventional pushing-coil, but it is sufficient for a successful treatment in the majority of cases [19]. N2BCA has also a cosmetic advantage, as it is absorbed over time and does not appear in diagnostic images [19].

The main disadvantage of glue is that its release is not fully controllable by the operator [19]. Cyanoacrylate varicocele embolization has been reported with some complications: the most common was the perforation of the ISV with contrast extravasation (5.8% of patients), with no increase in the need for reintervention [56]; other reported complications are glue migration into pulmonary circulation, glued catheter, phlebitis of pampiniform plexus and ISV and post-embolization pain [26,45,56,65,66].

Vanlangenhove et al. reported a late inflammatory reaction to the cyanoacrylates: during the week after embolization, 59% of patients reported some discomfort, which was in 35% at least a bearable pain [15]. Many studies report a technical success rate of 92% for glue embolization of male varicocele [56], with a lower recurrence rate (4.2%) than other embolic agents due to the penetration into the collaterals and a low technical failure rate (<1%) related to the use of a coaxial catheter system [15].

Percutaneous sclerotherapy of varicocele was first introduced by Porst et al. in 1984 and it could be considered the standard and most frequent percutaneous approach for MV [26,67]. In Europe, sodium tetradecyl sulfate 3% (STS), sodium morrhuate, dextrose, hydroxypolyethoxydocanol and polidocanol (Aetoxysclerol^®^ 1–2 or 3%, Kreussler Pharma, Paris, France) [68] are the most common sclerotic agents. The Sclerosing agent can be used alone or in combination with coils or balloon [19,25,45] and can be injected as a liquid or as foam [15]. Foam sclerotherapy has been considered to offer several advantages over traditional liquid sclerotherapy [57]. The foam has the advantage of a better distribution of the sclerosing agent on the endothelial surface and of multiple collateral branches, resulting in a more effective embolization [69].

The sclero-embolization technique consists of injecting a sclerosing agent, as a pure liquid or mixed with air as foam, into the distal portion of the ISV [15,25,45,69]. The sclerosing agent is prepared in foam by mixing the contents of two syringes, one containing the sclerosing agent and the other containing unfiltered room air, according to Tessari et al. [70]; some authors use a ratio of 1:1 (air and sclerosing), adding 2 mL of contrast media to create the foam [25,45], others use 70% of sclerosing agent and 30% of air [69]. Before injecting the sclerosing agent, a distal barrage near the external inguinal ring is mandatory to prevent the agent from penetrating into the pampiniform plexus during Valsalva maneuver, hence avoiding inflammation and thrombosis (thrombophlebitis) of the pampiniform plexus; for this purpose, some authors place coils at the level of the inguinal canal [45], while others use manual compression [15] or rubber band applied at the highest level of the scrotum, kept in place for 10 min after sclerosing injection and then released [69]. The sclerosing agent is administered under fluoroscopic guidance, through a catheter whose tip is placed in the most distal part of the ISV, at the level of the sacroiliac joint or at the lower edge of the ischiopubic ramus [15,45].

**Figure 3:**
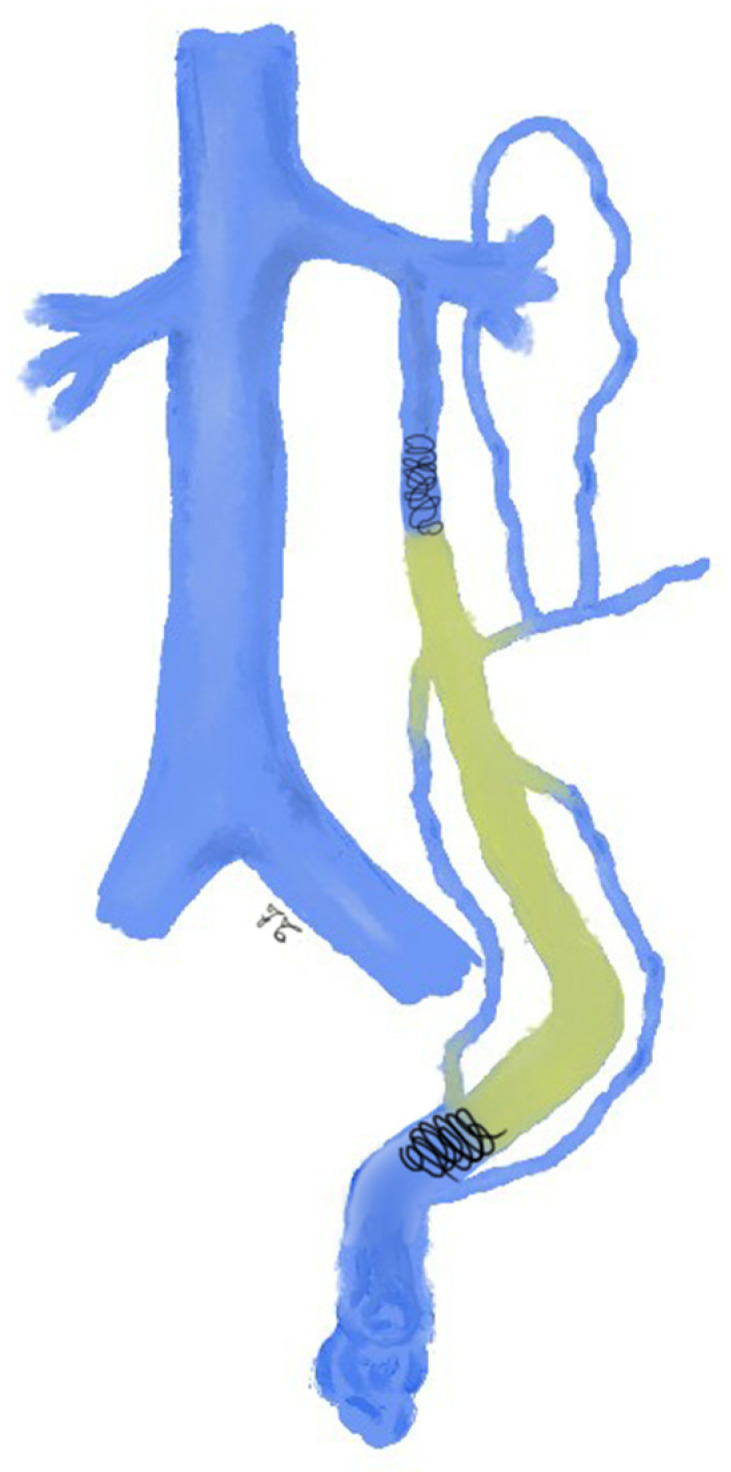
Drawing shows spermatic vein embolization with sclerosing agent: the sclerosant is administered through a catheter whose tip is placed in the most distal part of the ISV, at the level of the sacroiliac joint. Additional coils are deployed at the proximal part of the spermatic vein according to the sandwich technique.

During the injection, the patient should perform the Valsalva maneuver to prevent the sclerosing agent refluxing into the renal vein [15,45]. In case of other collateral veins originating below the sacroiliac joint, the catheter should be positioned at the level of their origin, to allow the extensive sclerotherapy of all collaterals [15,45]. Some authors suggest deploying additional coils at the proximal part of the spermatic vein according to the sandwich technique (Figure 3) [45].

**Figure 4:**
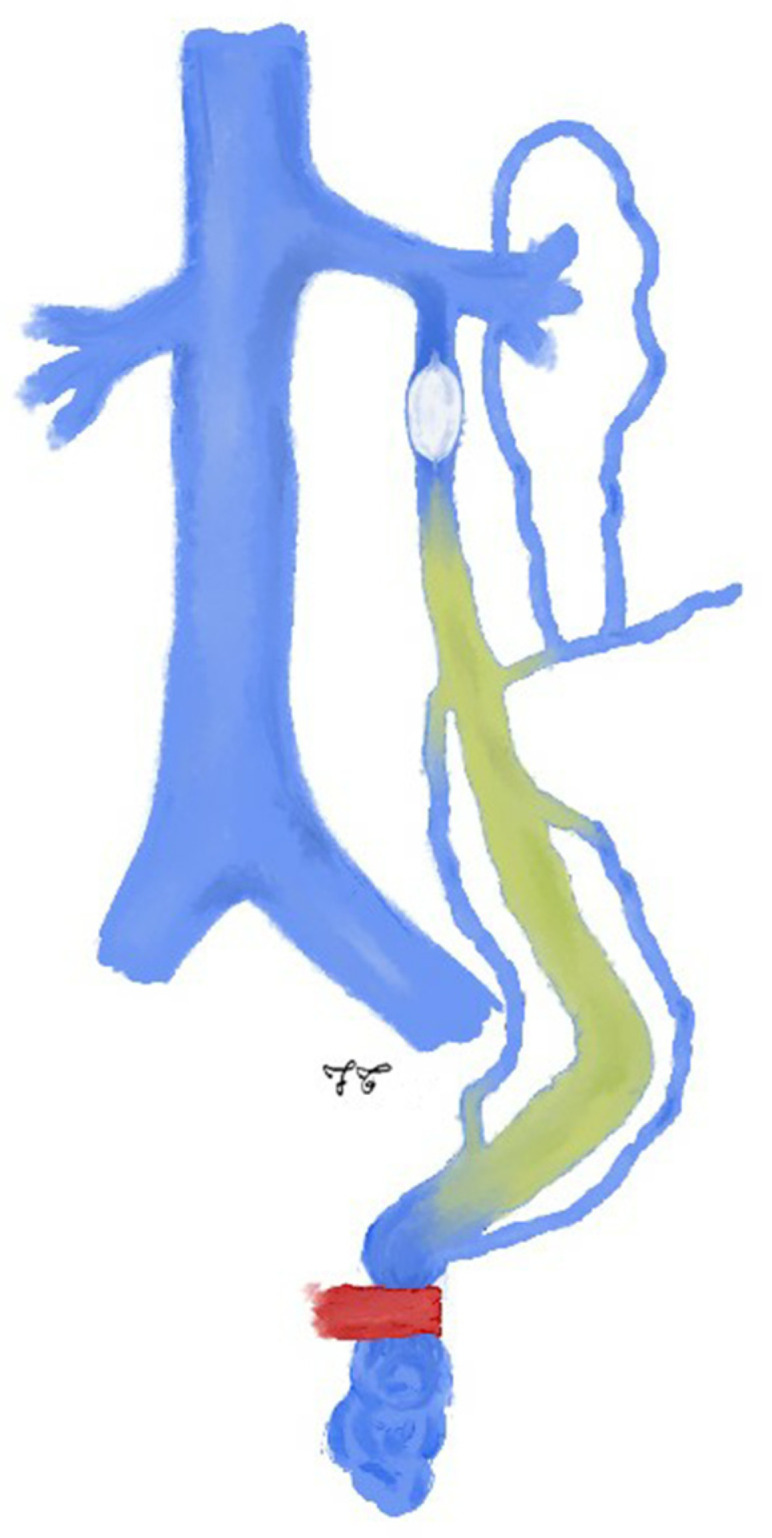
Drawing shows spermatic vein embolization with sclerosing agent according to occluding balloon technique: this technique refers to the use of a temporary proximal OB catheter in addition to distal barrage, to stop the retrograde blood flow. The sclerosing agent is injected through the OB catheter into the distal portion of the ISV.

Basile et al. propose a variation to traditional sclerosing technique, similar to the balloon-occluded retrograde transvenous obliteration (BRTO) technique for gastric varices treatment [71]. This technique, also known as “OB technique”, refers to the use of a temporary proximal OB catheter in addition to distal barrage, to stop the retrograde blood flow: the sclerosing agent is injected through the OB catheter into the distal portion of the ISV without any Valsalva maneuver (Figure 4). Through the OB technique, the sclerosing agent becomes more controllable during its injection and remains in constant contact, at highest concentration, with the vessel walls [69].

The advantage of Sclerosing agents is that they spread beyond the main gonadal vein through the collaterals, thus preventing possible recurrence [15,19,25,45], but their low viscosity and visibility might increase nontarget embolization [15]. Sclerotherapy improve testicular function and seminal parameters, increasing sperm density, motility and morphology [59,72,73,74], and also pregnancy rates (39%), as described by Gandini et al. [73].

A disadvantage of the sclerosing agents use, in case of free catheter injection, is that agents’ concentration into the vessels is related to the patient’s ability to maintain Valsalva maneuver; in cases where the patient is unable to hold a deep Valsalva or in patients with pain or sedation, the sclerosing agent can be diluted and so its effectiveness is decreased. To overcome this problem, OB technique could be used, as it does not require Valsalva maneuver and could be even more comfortable for patients [69].

Fever, epididymo-orchitis, testicular or groin swelling and hydrocele were observed in 1.9% of patients; these were the most frequently encountered complications, followed by spermatic vein rupture and extravasation (0.9%) and allergic reactions (0.2%) [56]. In case of manual compression used as distal barrage, pampiniform plexus thrombophlebitis was reported as the most common complication, due to an insufficient manual compression of the inguinal ring during injection, allowing the sclerosing to pass in the pampiniform plexus [25].

Scleroembolization of MV presents a technical success rate of 92.5% and a relapse rate of 11.03% [56]. Ali et al. reported that 94.9% of patients refer a complete resolution of pain or discomfort and in 97% of cases the resolution of testicular swelling or palpable “bag of worms”. Sclerotherapy with polidocanol presented higher pregnancy rates than surgical treatment options and has the advantage of no association with hydrocele [25]. Transcatheter embolization with sclerosing agent can be considered the most effective and safe procedure for treatment of MV and may be the preferable treatment option for patients with unilateral varicocele [23].

Vanlangenhove et al. tested Onyx as an embolic agent in ISV embolization and found that Onyx was efficient and indeed better tolerated in the post-embolization period, but there was an acute pain reaction during the injection in most patients. Patients’ discomfort and high radiation dose preclude, at the moment, Onyx clinical use [15].

The previous publications taken into consideration, the embolic materials used, the technical success rate, the recurrence rate and complications for male varicocele are summarized in Table 2.

## 4. Clinical Results in Pelvic Congestion Syndrome

Trans-catheter endovascular embolization technique allows a permanent vascular occlusion of the uterine and pelvic veins with multiple embolic agents; in literature, treatments have been reported with both liquid agents and solid agents such as coils and plugs, that can be used alone or in combination [75]. In general, the choice of the embolic agents is up to operators’ experience and preferences, since clinical and technical success rates are high for all of them. Endovascular treatment is essentially aimed at treating ectatic vessels; there is no agreement in the literature on how many vessels should.

**Figure 5:**
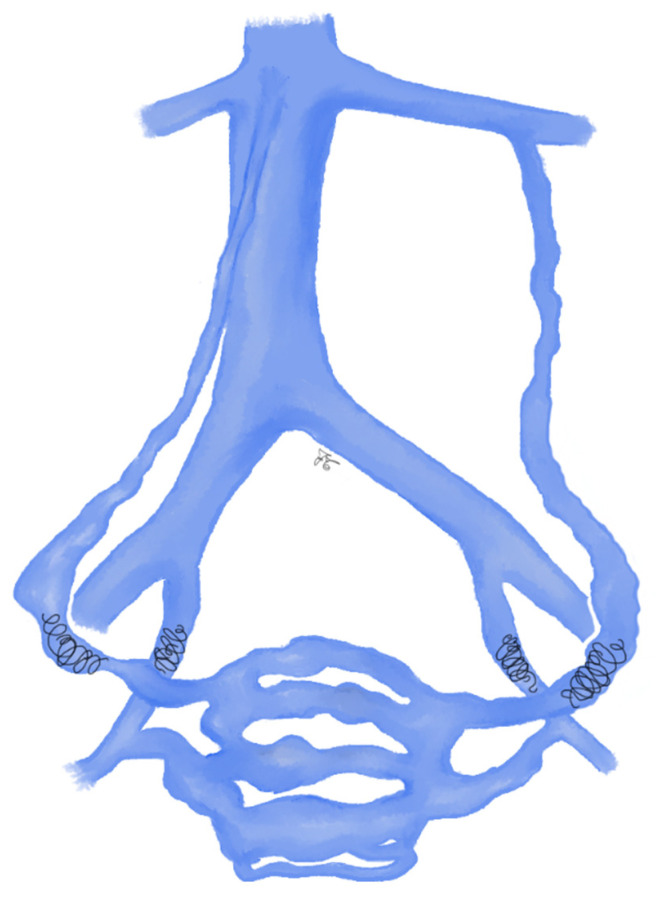
Drawing shows left and right ovarian veins and left and right hypogastric veins embolization with coils, as reported by Laborda et al. [76].

Regarding the technical aspects, some authors suggest that the release of coils should begin at the lower aspect of the ovarian vein, trying to avoid the occlusion of the deep pelvic plexus; stainless steel or fibered platinum coils of several sizes (4–20 mm) can be used (Figure 5) [76]. In expert hands, a technical success of 100% has been reported, and a recent systematic review reports that partial or total clinical improvement (evaluated with VAS score at follow-up) following coils embolization ranges from 82.1% to 100% [77].

The main complications are represented by migration of the coils (described in 1.9% of cases) followed by coil misplacement, vein perforation, local phlebitis, and re-canalization because of coil erosion; most adverse events are early, and no significant complications have been reported on prolonged follow-up [76].

The use of Amplatzer vascular plug (AVP) for embolization of the ovarian vein has been reported in the literature by Basile et al.; these authors used AVPs of 12 mm and 14 mm on a patient who was symptom-free after a nine-month follow-up; the authors reported that the positioning of these devices was relatively simple and fast, with possible reduction of radiation exposure [78]. Although this technique seemed to be promising, there are no other similar case reports to the best of our knowledge.

Gelfoam can be administered by freehand injection or with the aid of an occluding balloon (OB) angiographic catheter [35]. The target vein and the catheter should be pre-filled with iodinated contrast and later the foam should be slowly injected, replacing the contrast in the target vessel in order to ensure the perfect vessel coverage and reduce the reflux. Studies reported in the literature have always used gelfoam in combination with other embolizing agents (coils, glues), and therefore it is not possible to comment on the efficacy of gelfoam as the only embolizing agent in the treatment of PCS [77,79].

Liquid agents that have an application on PCS treatment are sclerosing agents (polidocanol, tetradecyl sulfate, sodium morrhuate), lipiodized oils, alcohol and glues.

Embolization with glues, possibly in association with coils, is a valid alternative in the treatment of PCS. Enbucrilate, also known as n-Butyl cyanoacrylate, n-BCA or NBCA, is a liquid embolic system, composed of cyanoacrylate, which is usually administered mixed with lipiodized oil to increase its radiopacity.

**Figure 6:**
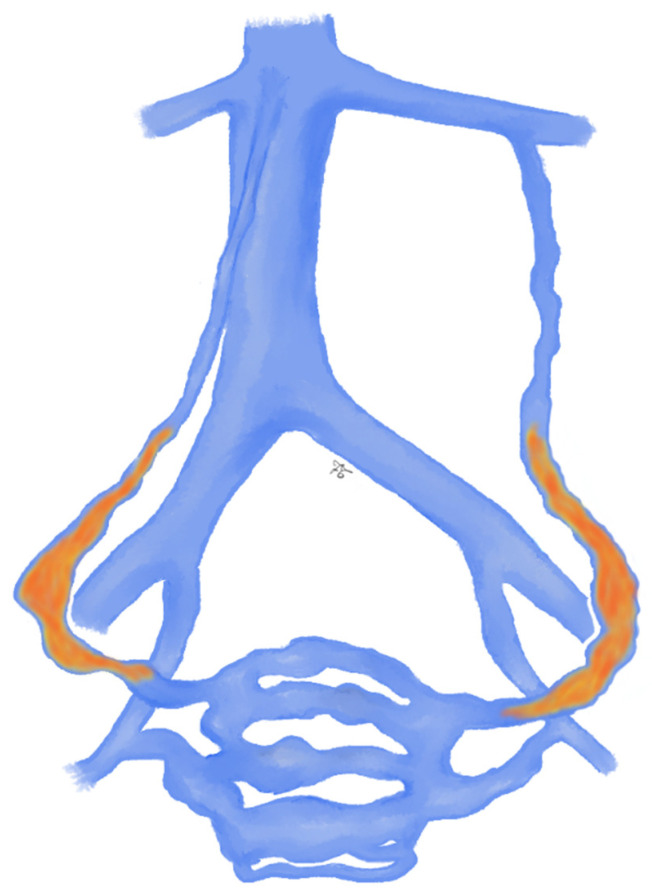
Drawing shows left and right ovarian veins embolization with glue: embolization should start from the distal portion of the ovarian vein at the level of the upper half of the sacroiliac joint, to include possible collateral branches.

NBCA embolization, alone or in association with coils, for the treatment of PCS was first described by Capasso et al. in 1993 [80]. As reported in literature, the procedure is performed in a one-day-clinic setting; the patient should be placed in a semi-upright position, in order to reduce the risk of glue migration into the systemic circulation, eventually performing a Valsalva maneuver [81]. Embolization should start from the distal portion of the ovarian vein at the level of the upper half of the sacroiliac joint, to include possible collateral branches (Figure 6) [80]. The technical success rate of embolization with NBCA is between 96.7% and 98%, with total or partial relief of symptoms in 57.9% and 15.8% of cases in follow-up, respectively [80,81]. No significant differences were found in symptom relief in patients with mono or bilateral disease, nor as a function of parity, and the presence of initial dyspareunia does not affect the results [81]. The main complications of this embolization technique are the glue migration into the pulmonary circulation, described in two cases by Maleux et al. and the perforation of the vessels caused by the guidewire, described in two cases by Capasso et al. [80,81].

Sclerosing agents have also been used in PCS: as far as we know, sodium tetradecyl sulfate and sodium morrhuate were used in reproducible studies with a sufficiently detailed protocol.

**Figure 7:**
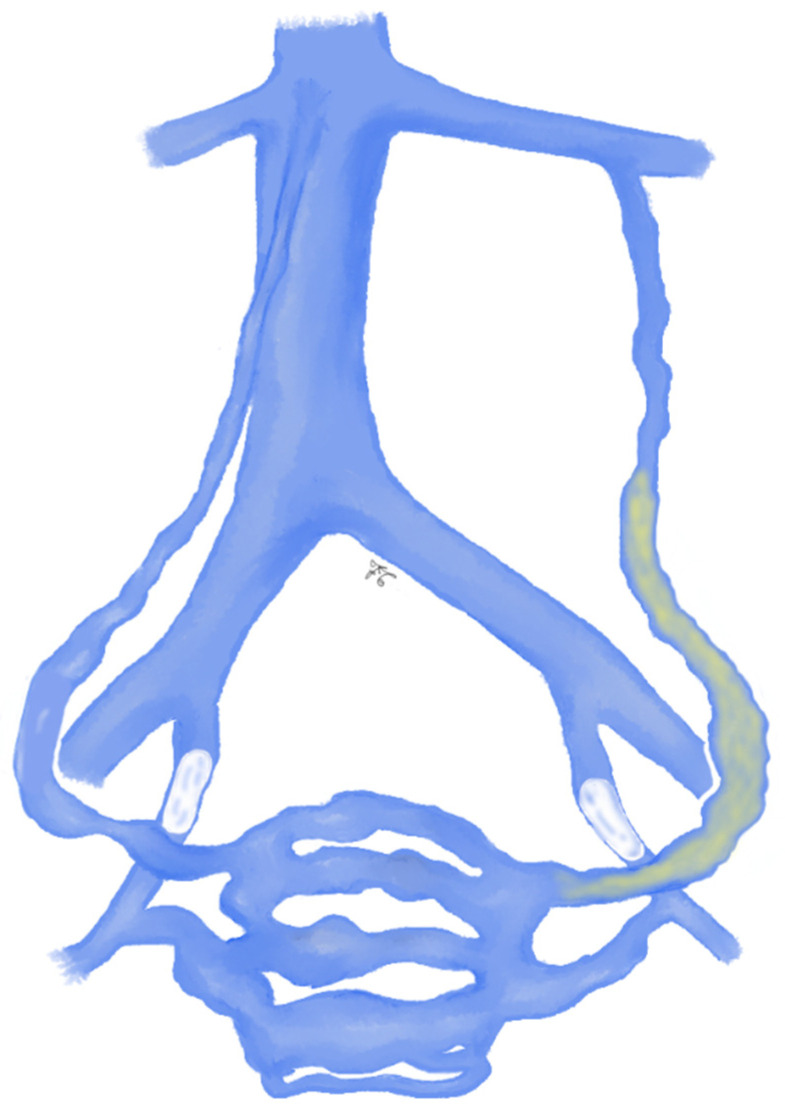
Drawing shows left ovarian vein embolization using the stop-flow foam sclerotherapy (SFFS) reported by Gandini et al. [82]: the sclerosing agent was injected into the pelvic vessels after having inflated the balloon catheter to occlude the major tributary vessels (hypogastric and/or ovarian veins) and excluding high-outflow venous collaterals.

Regarding the technique details, Gandini et al. reported that they performed stop-flow foam sclerotherapy (SFFS) using 20–40 mL of 3% STS (sclerosing agent), which was injected into the pelvic vessels after having inflated the balloon catheter to occlude the major tributary vessels (hypogastric and/or ovarian veins) and excluding high-outflow venous collaterals (Figure 7) [82].

This combined treatment (sclerosing agent and OB) was associated with an improvement of symptoms: pelvic and menstrual pain were significantly reduced in the follow-up up to one year, as well as urinary urgency and dyspareunia [82] in comparison to other previous studies, in which the OB was not used, that detected a clinical failure in 39% of cases and associated with pelvic varicosities with diameters greater than 5 mm [83]; the authors reported no significant complications related to the combined endovascular technique, but it was common for patients to experience colic-like pain soon after injection of the sclerosing agent [82].

**Figure 8:**
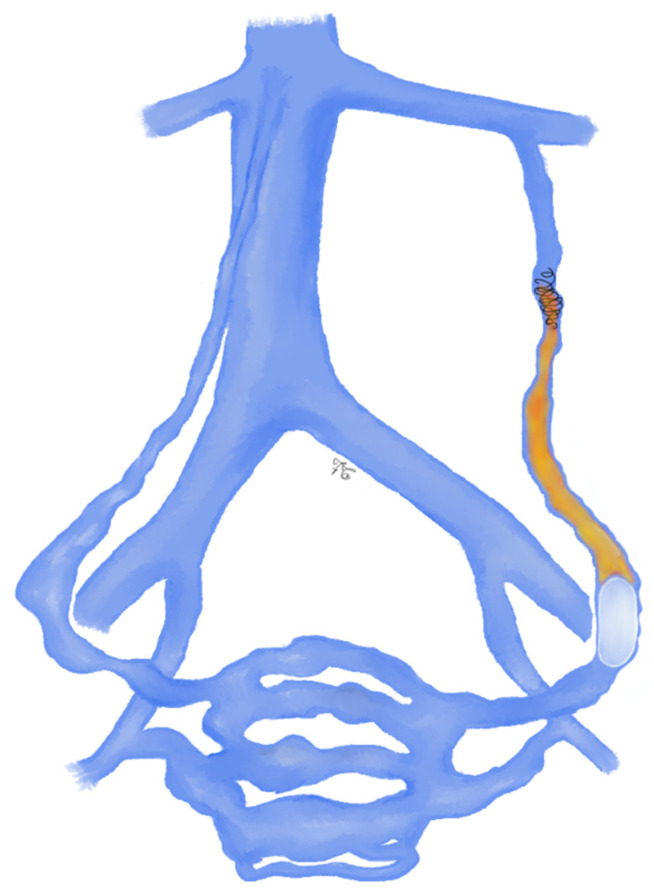
Drawing shows left ovarian vein embolization with sclerosing agent and coils: an occluding balloon is advanced to the lower third of the sacroiliac joint for the embolization of the gonadal vein; balloon insufflation was maintained for 5 min after the administration of sclerosant and the procedure was completed with the placement of metallic coils as reported by Meneses et al. [84].

Meneses et al. used sodium morrhuate as a sclerosing agent in a small cohort of women with PCS and relapsing varicose veins after surgery. In this study, the authors advanced the OB to the lower third of the sacroiliac joint or to the incompetent vein, respectively, for the embolization of gonadal or iliac veins; balloon insufflation was maintained for 5 min after the administration of sodium morrhuate and the procedure was completed with the placement of 14 cm length and 8 to 12 cm diameter metallic coils (Figure 8) [84]. The authors reported technical success in all patients, with significant improvement in venous scores clinical severity score (VCSS) and a visual analogue scale (VAS) for pain assessment; no major complications during or after the procedure were reported, but all treated patients experienced severe pelvic pain for approximately five minutes immediately after the administration of the sclerosing agent [84].

It is difficult to establish whether unilateral embolization has a different result than a bilateral approach, as the studies and the opinions are heterogeneous and divergent [77]; however, it seems that there is no statistically significant difference in performing a unilateral or bilateral procedure, in terms of clinical outcome [77]. The clinical success of combined interventions on ovarian, internal iliac and varicose veins appears to be lower than for interventions limited to ovarian and iliac veins [85]. Finally, parity does not appear to be associated with difference in outcome in terms of clinical success in follow-up, regardless of the agent used [81].

The previous publications taken into consideration, the embolic materials used, the technical success rate, the recurrence rate and complications for pelvic congestion syndrome are summarized in Table 3.

## 5. Conclusions

For both male varicocele and pelvic congestion syndrome, different and heterogeneous endovascular treatment techniques have been reported for the local application of liquid or embolic agents, in some cases also in combination (coils and sclerosant, occluding balloon and sclerosant).

Some studies presented in this narrative review of the literature are detailed to allow them to be replicated in daily clinical practice. Overall, endovascular techniques proved to be relatively well tolerated because they were less invasive and with shorter hospitalizations than traditional surgery (often one-day setting). For the treatment of male varicocele with embolizing agents and endovascular techniques, a rather variable technical success rate is reported in the studies, ranging from 70% to 100%, and recurrence rates of up to 16% of cases in the observational studies have been described: it should be noted that the recurrence rate is slightly higher than for surgical procedures, and this should be discussed with the patient undergoing the procedure. The treatment of PCS with endovascular embolism has a rather high success rate, reported between 96% and 100% in the series that we have reviewed, while the recurrence rate is highly variable according to the authors, ranging from 0% to 39% (worse results if only sclerosing agents are used)

The main complications reported in the literature, for both the treatment of male varicocele and PCS, are represented by the migration of embolizing media in distal sites, in collateral circulation or in the pulmonary circulation, but the consequences in all reported cases have been self-limiting.

In this scenario, the interventional radiologist becomes the main element of both the diagnostic and the therapeutic aspects.

## Figures and Tables

**Table 1:** This table summarizes embolic agents used in gonadal veins embolization.

Gonadal Veins Embolic Agents		
	Materials	Mechanism of Action
Solid Embolic Agents		
***Coils***	Platinum or stainless steel	Permanent Mechanical Thrombogenicity Platelets activation
***Vascular Plugs***	Disks of self-expanding nitinol mesh	Permanent Mechanical
***Balloons***	Detachable balloons, occluding balloons	Permanent Mechanical
Liquid embolic agents		
***Glues***	N-butyl-cyanoacrylate (NBCA)N-butyl-cyanoacrylate + methacryloxysulfolane, (NBCA-MS)	Polymerization in contact with blood Exothermic reaction Damage of the vascular endothelium
***Sclerosing agents***	Polidocanol Sodium tetradecyl sulfate (STS)Sodium morrhuate Ethanolamine oleate	Polymerization in contact with blood Venous wall damage Occlusion of the vessel
***Onyx^®^***	Ethylene-vinyl alcohol (EVOH)	Polymerization in contact with blood Solidification to a rigid cast

**Table 2:** This table summarizes the publications taken into consideration, the embolic materials used, the technical success rate, the recurrence rate and complications for male varicocele.

Author	Study Design	Embolic Agent	Patients	Technical Success	Recurrence Rates	Complications
Male Varicocele Embolization						
Ali et al. [25]	Observational study retrospective	Sclerosant	141	91.8%	16.10%	Contrast agent extravasation, and pampiniform plexus phlebitis (*n* = 1), temporary inguinal/ scrotal swelling (*n* = 1), temporary minimal groin hematoma, of temporary pain in the flanks (*n* = 1).
Basile et al. [69]	Randomized controlled clinical trial Prospective	Sclerosant + coils	90	75.6–93.4%	6.7–11.2%	Vein rupture with contrast leakage (*n* = 10), minor groin hematoma (*n* = 7)
Bechara et al. [20]	Comparative study Retrospective	Coils	41	95%	4.8%	NR
Bilrerio et al. [58]	Retrospective study	Coils vs. Glue	129	100% for glue, 99% for coils	11.54% with glue and 5.83% with coils	light testicular pain lasting 5 months after embolization with coils (*n* = 1)
Di Bisceglie et al. [72]	Non-randomized controlled study Prospective (treated group vs. control)	Sclerosant and coils	223	n/a	7.60%	acute abdominal pain (*n* = 2),2 spermatic cord inflammation (*n* = 2), vasovagal attack (*n* = 1)
Favard et al. [45]	Comparative study	Glue vs. Coils vs. sclerosant and coils	182	Only successful cases	11 vs. 13.2 vs. 6%	Pampiniform plexus phlebitis (*n* = 2), minor groin hematoma that resolved spontaneously (*n* = 4)
Gandini et al. [73]	Observational study Retrospective	Sclerosant	244	97.1%	3.6–6.7%	Allergic reaction (*n* = 2), retroperitoneal leakage of contrast medium (*n* = 4), short episode of fever (*n* = 2), testicular swelling that resolved (*n* = 2)
Gazzera et al. [59]	Observational study/controlled study Prospective	Sclerosant and coils	223	92.3%	16.5%	Acute abdominal pain (*n* = 2), Vasovagal attacks during administration of sclerosing agent that resolved spontaneously (*n* = 3) spermatic cord inflammation that resolved within days after medical therapy (*n* = 2)
Li et al. [74]	Observational study Retrospective	Sclerosant	58	100%	8.6%	NR
Puche-Sanz et al. [57]	Observational study Retrospective	Coils	154	95.5%	13.1%	Hydrocele (*n* = 7), femoral phlebitis (*n* = 1)
Reyes et al. [18]	Observational study Retrospective	Detachable balloons with or without “sandwiched” 70% dextrose and coils	59	90%	10%	Migration of a balloon to the lung (*n* = 1); nausea and vomiting (*n* = 2)
Vanlangenhove et al. [14]	Randomized controlled trial prospective	glue	83	70.7–83.3%	n/a	Acute allergic reaction immediately after embolization (*n* = 1)
Urbano et al. [19]	Observational study Retrospective	Glue	41	100%	0%	Moderate post- embolization pain (*n* = 7) that required oral analgesic treatment for 7–10 days
White Jr et al. [60]	Observational study Retrospective	Detachable balloon	70	NR	11%	migration of a balloon to the lung (*n* = 1)

**Table 3:** This table summarizes the publications taken into consideration, the embolic materials used, the technical success rate, the recurrence rate and complications for male pelvic congestion syndrome.

Author	Study Design	Embolic Agent	Patients	Technical Success	Recurrence Rates	Complications
Pelvic Congestion Syndrome Embolization						
Basile et al. [78]	Letter to the editor/case report	Sclerosant + AVP	1	100%	−	−
Capasso et al. [80]	Observational study retrospective	Glue and/or macrocoils	19	96.7%	NR	Perivulvar ovarian vein perforation (*n* = 2)
Gandini et al. [82]	Observational study retrospective	Sclerosant	26	100%	−	Colic-like pain after the injection of the sclerosing agent (*n* = 26)
Laborda et al. [76]	Observational study retrospective	Coils	202	100%	12.5%	Groin hematoma (*n* = 6), coil migration (*n* = 4), and reaction to contrast media (*n* = 1).
Maleux et al. [81]	Observational study retrospective	Glue and lipiodized oil Glue and microcoils (*n* = 1)	41	98%	NR	Migration of some fragments of glue (*n* = 2)
Meneses et al. [84]	Observational study retrospective	Sclerosant and coils	10	100%	−	No major complications, pelvic pain for 5 min after the sclerosant injection (*n* = 10)
Pieri et al. [83]	Observational study retrospective	Sclerosant	33	NR	39%	NR

NR= Not reported.

## Data Availability

Not applicable.
